# Automatic Detection of AMD and DME Retinal Pathologies Using Deep Learning

**DOI:** 10.1155/2023/9966107

**Published:** 2023-11-24

**Authors:** Latifa Saidi, Hajer Jomaa, Haddad Zainab, Hsouna Zgolli, Sonia Mabrouk, Désiré Sidibé, Hedi Tabia, Nawres Khlifa

**Affiliations:** ^1^Laboratory of Biophysics and Medical Technologies, Higher Institute of Medical Technologies of Tunis, University of Tunis El Manar, Tunis, Tunisia; ^2^Laboratory of Biophysics and Medical Technologies, National Engineering School Tunis, University of Tunis El Manar, Tunis, Tunisia; ^3^Department A, Hedi Raies of Ophthalmology Institute, Tunis, Tunisia; ^4^IBISC, University of Paris-Saclay, Univ Evry, Evry, France

## Abstract

Diabetic macular edema (DME) and age-related macular degeneration (AMD) are two common eye diseases. They are often undiagnosed or diagnosed late. This can result in permanent and irreversible vision loss. Therefore, early detection and treatment of these diseases can prevent vision loss, save money, and provide a better quality of life for individuals. Optical coherence tomography (OCT) imaging is widely applied to identify eye diseases, including DME and AMD. In this work, we developed automatic deep learning-based methods to detect these pathologies using SD-OCT scans. The convolutional neural network (CNN) from scratch we developed gave the best classification score with an accuracy higher than 99% on Duke dataset of OCT images.

## 1. Introduction

Nowadays, life expectancy has been greatly increased, but preserving vision is also essential to maintain a good quality of life. Vision loss is therefore an alarming problem for the world population. According to the report published by the International Agency for the Prevention of Blindness (IAPB), age-related macular degeneration (AMD), which affects adults over the age of 50, is the third leading cause of visual impairment and irreversible blindness in the world population. This means that 8.1 million people worldwide have vision loss due to AMD not treated in time [1].

Among the leading causes of vision loss in young adults in developed countries is diabetic macular edema (DME), which is a major complication of diabetic retinopathy in diabetic patients. It is also the leading cause of blindness in people under the age of 50. These pathologies are sometimes misdiagnosed or diagnosed late. This can lead to permanent and irreversible vision loss. Thus, the cost incurred will be very high [2].

Optical coherence tomography (OCT) imaging is the widely used technique for detecting many eye diseases such as AMD and DME. It provides a 3D cross-sectional view of the human eye. In addition, it can visualize all layers of the retina at a higher resolution. Spectral domain-optical coherence tomography (SD-OCT) is a noninvasive imaging method that can be used in vivo [3], based on low coherence interferometry, like ultrasound, except that IR light (photon laser/810 nm) replaces sound. It analyzes the spectrum of the reflected light signal improving the speed of the examination by a factor of 100 compared to traditional time domain OCT (TD-OCT) where retinal depth information is obtained after a longitudinal translation in time of a reference beam. OCT allows the precise analysis of macular abnormalities and is essential for the follow-up of these abnormalities. It has become a major tool for the detection of retinal and pigment epithelial anomalies and especially choroidal neovessels. The technique does not involve any radiation for the patient.

Early detection and treatment of AMD and DME will prevent vision loss, save money, and provide a better quality of life for individuals. There are various classical methods such as retinal layer segmentation [4] and recent methods such as deep learning that has been used to help ophthalmologists to detect eye diseases more easily. Many papers have been published in this field based on OCT scan data [5, 6]. However, some of them can be confusing in their predictive results and require specialist intervention. In fact, from these existing studies, we note that each approach has its own advantages and disadvantages. Indeed, the majority of these methods are used in a well-defined context. So, there is no perfect method that predicts retinal decease using OCT images. Deep neural networks have been the most accurate method for the automatic diagnosis of retinal diseases [7–9]. They have received more attention and progress in medical image analysis [10, 11].

In this work (work carried out in the OCTIPA project (CMCU 23G1418), as part of the PHC-Utique program managed by the CMCU of the French Ministry of Europe and Foreign Affairs and the Tunisian Ministry of Higher Education and Scientific Research), the main objective is to detect retinal pathologies from SD-OCT images. Indeed, we proposed a classification model that can detect AMD and DME without any intervention from clinical experts. In fact, the costs for people suffering from these diseases can be enormous. Therefore, early detection of retinal diseases is challenging because they often start asymptomatic and develop over time.

The model should provide the ability to automatically detect pathology with high accuracy to predict image classes as normal or infected.

The rest of the paper is structured as follows: Section 2 presents related works, Section 3 describes the proposed method, and finally, the experimental results are discussed in Section 4.

## 2. Related Work

Over the past 20 years, advances in ophthalmic image processing have enabled the development of automated diagnostic systems for many diseases such as diabetic retinopathy, AMD, macular hole, and DME. These diagnostic systems which automate the process of detecting eye diseases have attracted a lot of attention from clinicians and researchers. They do not only ease the workload of clinicians by providing objective opinion and valuable information but also offer early detection and easy patient accessibility.

In the following, we detail some recent work on deep learning-based CAD systems for OCT image classification.

Bhowmik et al. [12] proposed a transfer learning-based method using the two pretrained CNN architectures, VGG16 (23 layers) and InceptionV3 (159 layers), to predict eye pathologies from OCT images.

Before the implementation of the model, the authors applied a data preprocessing step by resizing the images. Then, they removed the “fully connected” layer for both models, keeping the convolution layer, and replace it by a polling layer followed by the flattening layer. The Softmax activation function was used, and as an optimizer, the authors used Adam and RMSProp with a learning rate of 0.0005.

The proposed approach was trained and validated on a dataset of 4000 OCT images labeled in 4 categories, normal, CNV, DME, and drusen, of which 80% (3200 images) were used for training and 20% (800 images) for testing. The experimental results indicate that the proposed method achieved an accuracy of 94%.

Yang et al. [13] presented an end-to-end weakly supervised convolutional neural network (WCNN) model to detect AMD pathology and localize its lesions in OCT images. Yang et al. proposed a CNN architecture based on a backpropagation algorithm called expressive gradients (EG). This one is generated from the integrated gradient (IG) algorithm. The authors proved that the proposed EG algorithm outperforms both the IG algorithm in localization accuracy and existing object detection methods in classification accuracy.

Their proposed approach was validated on 10,100 clinical OCT images with 3 classes, normal, dry AMD, and wet AMD, of which 9575 were used for training and 525 for testing. The validation indicates that the proposed method resulted in a test accuracy of 94.86% and a validation accuracy of 96.05%.

Wang D. and Wang L. [14] proposed a method for automatic detection of two pathologies (DMO and AMD) based on deep learning. They used two public datasets: the first one is provided by Srinivasan et al. and is obtained on Duke, and the second one is provided by Rasti et al. and is obtained on Kaggle from Noor Eye Hospital in Tehran. Wang D. and Wang L. evaluated the performance of the following pretrained CNN models: CliqueNet, DPN92, DenseNet 121, ResNet 50, ResNext101, VGG16, VGG19, and InceptionV3.

They used 3231 images from the first dataset and 5084 images from the second one. Each one was divided into 80% for learning and 20% for testing.

The experimental results indicate that DPN and CliqueNet achieved the best accuracy values including 99.6% and 99% on the first base and 95.8% and 98.6% on the second base, respectively.

Vaghefi et al. [15] demonstrated that high diagnostic accuracy can be achieved when deep learning combines analysis of multimodal OCT, OCT-A (OCT angiography), and CFP (color fundus photographs) images. The authors presented a CNN model based on the Inception-ResnetV2 architecture.

Each image modality was initially passed through the resizing layer, which was followed by 3 repetitions of convolution2D layer, batch normalization, and a RELU activation layer. Then, the three separate modalities were concatenated using a global pooling layer and then passed to the Inception-ResnetV2 model. The proposed approach of combining imaging modalities into a single “multimodal” CNN was used on data from 75 participants grouped into three categories: young healthy, elderly healthy, and patient with dry intermediate AMD. The results indicate that this method gave an accuracy of 99.8%, allowing the identification of both aging and disease with a high sensitivity and specificity of about 100% for each class.

Karri et al. [16] proposed a model based on the pretrained GoogLeNet model to classify OCT images into 3 classes: AMD, DME, and normal. They exploited for their work a dataset provided by Srinivasan et al. and obtained from Duke University of 15 subjects with AMD, 15 subjects with MDD, and 15 healthy subjects.

The authors adapted the pretrained model by “fine-tuning” after preprocessing the OCT images by the BM3D filter. The experimental results show that their method achieved an accuracy equal to 96%.

In Table 1, we summarize some relevant works.

## 3. Methods

### 3.1. Dataset

In this work, we implemented and tested our CNN models on a public dataset and on a private one. The public dataset was provided by Srinivasan et al. and was acquired using SD-OCT imaging (Heidelberg Engineering Inc. in Germany) at Duke University, Harvard University, and the University of Michigan. The dataset includes 45 SD-OCT volumes: 15 normal, 15 AMD, and 15 DME patients [17].

All volumes contain multiple B scans that range from 31 to 97 with a resolution of 496 × 768 pixels that we had grouped into 706 AMD, 1101 DME, and 1405 normal.

The other database used in this work is a Tunisian database containing 934 OCT images. These images were taken using Spectralis OCT Heidelberg machine at the Hedi Rais Hospital (http://www.santetunisie.rns.tn/), including three categories: AMD (538 images), DME (272 images), and normal (124 images).

### 3.2. Proposed Method

In this paper, we aim to build a model that allows to identify AMD and DME from OCT scan data with a higher accuracy without any human intervention. Firstly, we use the transfer learning using pretrained models. Next, we build a bilinear CNN using the pretrained models previously used in fine-tuning, and finally, we introduce a new CNN model architecture from scratch to classify OCT images.

**Figure 1:**
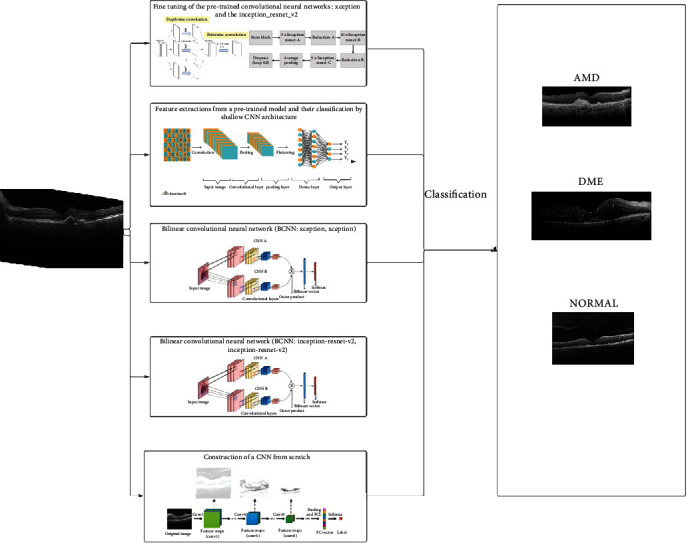
Proposed method.

The proposed method is illustrated in Figure 1.

#### 3.2.1. Preparation and Preprocessing of Data

Before applying the pretrained CNN model, a data preprocessing step is essential to improve the deep learning and the performance of the results. Hence, the OCT volumes were routed in three steps:

- Data balancing: first, we balanced the three database classes used. In fact, we increased the number of images using the following effects, randomly applied on the input set: data normalization, rotation, shift in width and height, shear, zoom, mirror effect, and fill empty pixels by neighbors' values (fill mode=”nearest”) to have the same original size after shifts and rotation

**Figure 2:**
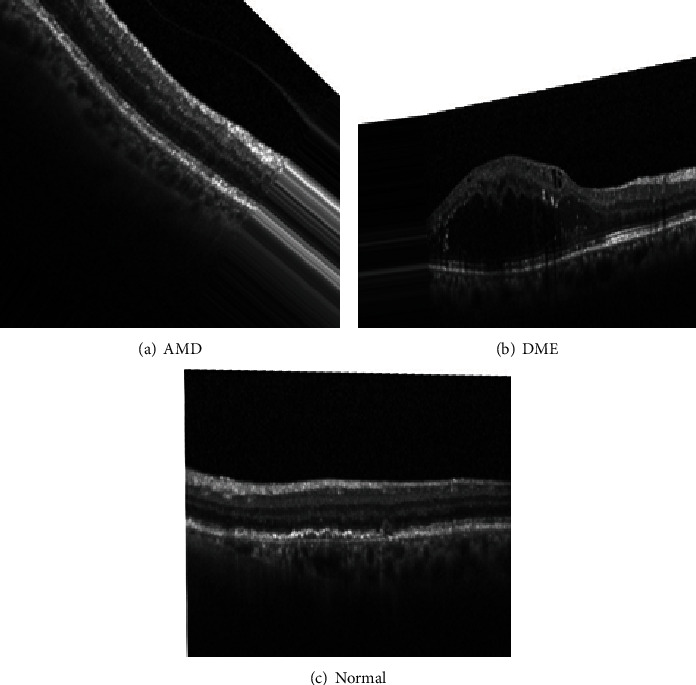
Three sample images derived from the data augmentation of the Tunisian dataset.

The application of these transformations gave us additional images like the ones shown in Figure 2, so the database becomes balanced and ready for use.

(ii) ROI and image resizing: ROI selection (region of interest) in the OCT images was made; then, an image resizing was done
(iii) Data splitting: the dataset was meticulously partitioned into training and test sets to optimize the learning process. Specifically, 70% of the images from each dataset were randomly allocated to the training set, allowing the models to learn from the inherent patterns and underlying features in the images. Additionally, a validation set was created from a portion of the training data, acting as a checkpoint to monitor the model performance on unseen data and prevent overfitting on the training set. The remaining 30% of images were set aside and reserved as the test set

#### 3.2.2. OCT Image Classification Based on Transfer Learning

Transfer learning mainly consists in applying the knowledge acquired from a pretrained CNN model (which was created for a source task) to a similar target task. It allows us to avoid reinventing a CNN from scratch, and it helps us to create AI applications in a very short time.

To use transfer learning, we should choose a pretrained model and then fine-tuning the model according to the classification problem to be studied or extract features from an intermediate layer and inject them into another CNN.

#### 3.2.3. Fine-Tuning of the Pretrained Convolutional Neural Networks

We used the two pretrained CNNs: Xception and the Inception_Resnet_v2:

- Xception [18]: it is a CNN architecture, inspired by Inception, and it is characterized by depth-separable convolution blocks with shortcuts between them as in ResNet. A depth-separable convolution can be understood as an Inception module with a maximum number of rounds. This model consists of 71 layers, among them 36 convolution layers. It is trained on more than a million images from the ImageNet database
- Inception_Resnet_v2 [19]: it is the second generation of Inception convolutional neural network architectures which notably uses batch normalization. It includes other changes like dropping dropout and removing local response normalization. This is a hybrid CNN architecture between “Inception Net” and “Residual Net.” It is the state-of-the-art pretrained model on more than one million images of ImageNet to perform 1000-category classification. This model has 165 layers in depth. It is downloadable from Keras API with ImageNet weights

We modified the last layer of the pretrained CNNs (Xception and Inception-ResnetV2) to adapt them to our classification case. We added a global average pooling layer “GAP” and a dropout layer “Dropout” to avoid overlearning. We added at the end the “Softmax” function with the number of outputs equal to 3 to have the predicted probability for each class.

*(1) Feature Extractions from a Pretrained Model and Their Classification by Another CNN*. Pretrained convolutional neural networks contain more information in the middle convolution layers than in the last ones. The principle of this idea is to import the architecture (Xception or the Inception-ResnetV2) and then look for the intermediate convolution layer that gives more features, from which we took the features. Then, we put them in another shallow CNN architecture.

Table 2 shows the structure of the layers of the created CNN.

For the “Xception” model, we extracted the features from the intermediate layer “block13_sepconv2_bn” which is the sixth layer of the penultimate block of separable architecture. This constructed neural network will be trained with randomly initialized weights to better learn the features extracted from the “block13_sepconv2_bn” layer of the Xception to improve the classification accuracy of the OCT images that we found with fine-tuning.

For the “Inception-ResnetV2” model, we extracted the features from the intermediate layer “block8_1_ac.” The extracted features (feature map), output from the Inceptionresnetv2 layer, are the input of the CNN architecture described in Table 2 with randomly initialized weights so that the model learns better and gives a higher classification accuracy of OCT images than the fine-tuning method.

#### 3.2.4. OCT Image Classification Based on Bilinear Convolutional Neural Network (BCNN)

The bilinear convolutional neural network (BCNN) is an architecture based on the combination of two CNN models, whether the two architectures used are different or identical. The principale of the BCNN consist to concatenate the characteristics extracted from each last convolution layer of the two CNN models [20].

More simply, when using an image A, as an input, this image passed through two different CNNs, and a feature map is then generated after applying several pooling and nonlinear transformations. These features are concatenated to make the model more reliable.

#### 3.2.5. Construction of a CNN from Scratch

We have created a CNN model from scratch, using the PyTorch Library. We gave the name “OCTorch-Net” for our created CNN model. The details of this model are given in Table 3.

Creating this CNN model with fewer layers than the pretrained CNN networks (Xception and Inception-ResnetV2) allows us to reduce the number of layers compared to the pretrained models. Indeed, the built CNN architecture is composed of a small number of layers, 8 convolution layers, only 3 MaxPooling layers, one flatten layer, one dropout layer, and one dense layer. This reduction of the number of layers of the network allows us to reduce the use of hardware systems (CPU/GPU) and the minimization of the learning time of the OCT image characteristics.

## 4. Results

### 4.1. Evaluation Metrics

#### 4.1.1. Accuracy

The accuracy allows to know the proportion of good predictions compared to all the predictions.

(1) $$\begin{array}{c} \text{Accuracy }\left(\text{ACC}\right)=\frac{\text{TP}+\text{TN}}{\text{TP}+\text{TN}+\text{FP}+\text{FN}}, \end{array}$$

where TP is the number of positive correct results in the dataset, TN is the number of negative correct results, FP is the number of false positive results, and FN is the number of false negative results.

#### 4.1.2. Precision

In the simplest sense, precision is the ratio of true positives to all positives.

(2) $$\begin{array}{c} \text{Precision}=\frac{\text{TP}}{\left(\text{TP}+\text{FP}\right)}. \end{array}$$

#### 4.1.3. Recall

Recall is a measure of our model correctly identifying true positives.

(3) $$\begin{array}{c} \text{Recall}=\frac{\text{TP}}{\left(\text{TP}+\text{FN}\right)}. \end{array}$$

#### 4.1.4. *F*1 Score

*F*1 score is a machine learning evaluation metric that combines precision and recall scores.

(4) $$\begin{array}{c} F1\text{ score}=\frac{2*\text{precision}*\text{recall}}{\left(\text{precision}+\text{recall}\right)}. \end{array}$$

### 4.2. Experimental Results

After preprocessing the balanced data and creating the CNN model, the hyperparameters used for all the models is the optimizer adam', the evaluation metric is validation accuracy, and the loss function is categorical_crossentropy.

The batch size is fixed as 128, and the number of epochs is equal to 30 for all models; expect the model built from scratch “OCTorch-Net”, we did only 20 epochs.

First, we used the following pretrained CNN models: Xception and InceptionResnetV2.

The results obtained on the Duke dataset are detailed in Table 4.

**Figure 3:**
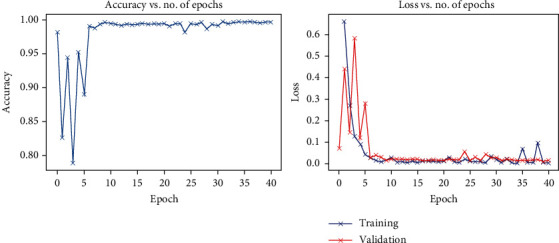
The performance of “OCTorch-Net” on the public database “Duke.”

Also, Figure 3 presents the evolution of the accuracy and the loss function for the model built “OCTorch-Net” on the public database “Duke.”

The results obtained on the Tunisian dataset are detailed in Table 5.

**Figure 4:**
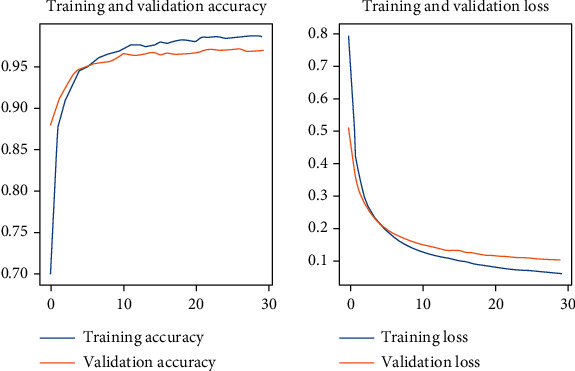
The performance of BCNN model (Xception, Xception) on the Tunisian dataset.

In addition, Figure 4 presents the evolution of the accuracy and the loss function for the BCNN model (Xception, Xception) on the Tunisian dataset.

## 5. Discussions

Deep learning models for retinal disease detection on OCT images have received much attention in many research fields such as medical analysis and computer-aided diagnosis (CAD). The suitability of deep learning methods depends simultaneously on the design of the model and its adaptation. In this work, we propose a new deep learning model applied to a medical domain with fewer processing treatment.

The results found show the importance of using deep leaning-based methods to detect retinal pathologies.

In fact, the pretrained CNN models gave significant performance when we extracted the features from a hidden layer and introduced them into another CNN architecture.

Also, the BCNN proved its importance to improve the results of a CNN. Indeed, by merging several features from several CNNs, the classification rates became more important, since the feature matrices will be richer in information. However, this introduces a complexity in the number of parameters used. The use of a CNN from scratch has allowed to overcome this problem, since the number of parameters is lower, although the results found are very satisfactory and even exceed the results obtained by fine-tuning of standard architectures. This new architecture OCTorch-Net can be validated on a larger number of data to be integrated in a real clinical context.

In fact, the OCTorch-Net is very effective for the prediction of the two retinal pathologies with an accuracy equal to 99.68% thanks to data augmentation, which generated an acceptable number of images. But, we are working currently on obtaining a large Tunisian database which will allow us to better enhance the reliability of the results and to test the models on a large number of data.

In Table 6, we present a comparison of the performances of the proposed methods and two methods from the literature.

It is clear that the proposed system achieved high classification accuracy and prediction ability of AMD and DME diseases compared to other state-of-the-art methods.

The results obtained prove that our architectures surpass most methods proposed in the literature. In particular, the new architecture has given excellent results that can be used in internal regions that suffer from a lack of specialized doctors.

## 6. Conclusion

We generated a deep CNN-based method to address the problems of multiclass classification of retinal pathologies (AMD, DME, and normal), which are the direct causes of vision problems, leading to total blindness in adulthood. Therefore, to avoid vision problems, our method presents an automated system to detect patients with macular edema or age-related macular degeneration using OCT scans because it is the common imaging method applied in ophthalmology in most countries of the world.

In fact, on Duke dataset the from scratch CNN “OCTorch-Net” gave the best classification score with an accuracy of 99.68%, while on Tunisian dataset, the BCNN model (Xception, Xception) gave the best performance with an accuracy of 98.56%.

We believe that the method can be further improved by adding spatial attention modules whose goal is to focus on the most information-rich regions that may be more significant than other regions.

## Figures and Tables

**Table 1:** Relevant works.

Reference	Pathology	Dataset	Preprocessing	Used CNN	Classifier	Result Val_accuracy
Bhowmik et al. [12]	DME, drusen, CNV, and normal	Kaggle dataset: retinal OCT images	Resizing	InceptionV3 and VGG16	Softmax	VGG16: 91.6% InceptionV3: 92%

Yang et al. [13]	Normal, dry and wet AMD	Local datasets	—	CNN (EG)	Softmax	96.05%

Wang D. and Wang L. [14]	AMD, DME, and normal	Dataset 1: SERI Dataset 2: Duke	Filtering: FastNIMeans+bilateralFilter	VGG16 VGG19 InceptionV3 CliqueNet DPN DenseNet ResNet ResNext		Dataset 1: VGG16: 91.6% VGG19: 98.2% InceptionV3: 92.7% CliqueNet: 99% DPN: 99.6% DenseNet: 98.7% ResNet: 98.7% ResNext: 97.3% Dataset 2: VGG16: 86.3% VGG19: 95.1% InceptionV3: 85.3% CliqueNet: 98.6% DPN: 95.8% DenseNet: 94.2% ResNet: 95.2% ResNext: 94.8%

Vaghefi et al. [15]	Normal_Young, Normal_Old, and dry AMD	75 participants	Resizing	Inception-ResnetV2	Softmax	99.8%

Karri et al. [16]	MD, DME, and normal	Duke	Denoising using BM3D+(flattening)	GoogLeNet	SVM	96%

**Table 2:** Description of the created CNN and its layers.

#	Layers	Values
1	Convolution2D	Number of filters: 32 Kernel size: 3^∗^3 Activation function
2	Dropout	0.2
3	Dense	4608, activation function: ReLu
4	Dense	128, activation function: ReLu
5	Dropout	0.2
6	Dense	64, activation function: ReLu
7	GlobalAveragePooling2D	—
8	Dropout	0.5
9	Dense	3, activation function: Softmax

**Table 3:** Description of the created CNN and its layers.

Blocks	Layers	Values
Conv_bloc 1	Convolution2D BatchNormalization2D Activation Function	Number of filters: 64 Kernel size: (3,3)
	Convolution2D	- ReLu Number of filters: 128 Kernel size: (3,3)

Conv_bloc 2	BatchNormalization2D Activation Function MaxPooling2D Convolution2D	- ReLu Kernel size: (2,2) with stride: 2 Number of filters: 128 Kernel size: (3,3)

Res_1	BatchNormalization2D Activation Function Convolution2D	- ReLu Number of filters: 128 Kernel size: (3,3)
	BatchNormalization2D Activation Function Convolution2D	- ReLu Number of filters: 256 Kernel size: (3,3)

Conv_bloc 3	BatchNormalization2D Activation Function MaxPooling2D Convolution2D	- ReLu Kernel size: (2,2) with stride: 2 Number of filters: 512 Kernel size: (3,3)

Conv_bloc 4	BatchNormalization2D Activation Function MaxPooling2D Convolution2D	- ReLu Kernel size: (2,2) with stride: 2 Number of filters: 512 Kernel size: (3,3)

Res_2	BatchNormalization2D Activation Function Convolution2D	- ReLu Number of filters: 512 Kernel size: (3,3)

MaxPool	BatchNormalization2D Activation Function AdaptiveMaxPooling2D Flatten	- ReLu - -

Bloc_classification	Dropout Dense Activation Function	0.2 512 Linear

**Table 4:** Obtained results on the Duke dataset.

Model	Accuracy	Precision	Recall	*F*1 score
Fine-tuning of Xception	96.83%	96.66	97%	97%
Feature extraction from “sepconv2_bn”+CNN	98.02%	98.33	98%	97.66%
BCNN (Xception, Xception)	97.84%	97%	97%	97%
Fine-tuning of Inception-ResnetV2	93.43%	93.66%	93.33%	93.33%
Features extraction from “block8_1_ac”+CNN	97.70%	98%	97.66%	97.66%
BCNN (Inception-ResnetV2, Inception-Resnet-V2)	95.55%	95%	95%	95.66%
The from scratch “OCTorch-Net”	99.68%	99%	96%	97%

**Table 5:** Obtained results on local dataset.

Model	Accuracy	Precision	Recall	*F*1 score
Fine-tuning of Xception	96.77%	96.66%	97%	97%
Features extraction from “sepconv2_bn”+CNN	98%	94.66%	94.66%	94.66%
BCNN (Xception, Xception)	98.56%	96.33%	96.66%	95%
Fine-tuning de Inception-resnet-v2	96.11%	95%	95%	95%
Features extraction from “block8_1_ac”+CNN	97.12%	98.33%	98%	98%
BCNN (Inception-ResnetV2, Inception-ResnetV2)	96.44%	98.66%	99%	98.33%
The from scratch “OCTorch-Net”	97.65%	96%	95.6%	95.8%

**Table 6:** Comparison of the results with some state of the art methods.

	CNN	Preprocessing	Accuracy
Karri et al.	GoogLeNet	BM3D	91.3%

Wang et al.	VGG16	FastNIMeans bilateralFilter	91.6%
	InceptionV3	FastNIMeans bilateralFilter	92.7%
	VGG19	FastNIMeans bilateralFilter	98.2%

Proposed architectures	Xception	ROI	96.83%
	Xception+feature extraction from middle layer	ROI	98.02%
	Inception-ResnetV2	ROI	93.43%
	Inception-ResnetV2+feature extraction from middle layer	ROI	97.70%
	BCNN (Xception, Xception)	ROI	97.84%
	BCNN (Inception-ResnetV2, Inception-ResnetV2)	ROI	95.55%
	The from scratch “OCTorch-Net”	Without preprocessing	99.68%

## Data Availability

We used a public dataset cited in P. P. Srinivasan, L.A. Kim, P.S. Mettu, S.W. Cousins, G.M. Comer, J.A. Izatt, and S. Farsiu, “Fully automated detection of diabetic macular edema and dry age-related macular degeneration from optical coherence tomography images”, BioMedical Optics Express, 5(10), pp. 3568-3577, 2014.
